# Natural and Enantiopure Alkylglycerols as Antibiofilms Against Clinical Bacterial Isolates and Quorum Sensing Inhibitors of *Chromobacterium violaceum* ATCC 12472

**DOI:** 10.3390/antibiotics10040430

**Published:** 2021-04-13

**Authors:** Klauss E. Chaverra Daza, Edelberto Silva Gómez, Bárbara D. Moreno Murillo, Humberto Mayorga Wandurraga

**Affiliations:** 1 Posgrado Interfacultades de Microbiología, Facultad de Ciencias, Universidad Nacional de Colombia, Av. Carrera 30 # 45-03, Edif. 224, Bogotá 11011, Colombia; kchaverra@unal.edu.co; 2 Grupo de Productos Naturales Vegetales Bioactivos y Química Ecológica, Laboratorio de Asesorías e Investigaciones en Microbiología, Departamento de Farmacia, Facultad de Ciencias, Universidad Nacional de Colombia, Av. Carrera 30 # 45-03, Edif. 450, Bogotá 11011, Colombia; esilvag@unal.edu.co; 3 Grupo de Productos Naturales Vegetales Bioactivos y Química Ecológica, Departamento de Química, Facultad de Ciencias, Universidad Nacional de Colombia, Av. Carrera 30 # 45-03, Edif. 451, Bogotá 11011, Colombia; bdmorenom@unal.edu.co

**Keywords:** antimicrobial resistance, natural alkylglycerols, ether lipids, 1-*O*-alkyl-sn-glycerols, antibiofilm activity, quorum sensing inhibition

## Abstract

Resistance mechanisms occur in almost all clinical bacterial isolates and represent one of the most worrisome health problems worldwide. Bacteria can form biofilms and communicate through quorum sensing (QS), which allow them to develop resistance against conventional antibiotics. Thus, new therapeutic candidates are sought. We focus on alkylglycerols (AKGs) because of their recently discovered quorum sensing inhibition (QSI) ability and antibiofilm potential. Fifteen natural enantiopure AKGs were tested to determine their effect on the biofilm formation of other clinical bacterial isolates, two reference strains and their QSI was determined using *Chromobacterium violaceum* ATCC 12472. The highest biofilm inhibition rates (%) and minimum QS inhibitory concentration were determined by a microtiter plate assay and ciprofloxacin was used as the standard antibiotic. At subinhibitory concentrations, each AKG reduced biofilm formation in a concentration-dependent manner against seven bacterial isolates, with values up to 97.2%. Each AKG displayed QSI at different levels of ability without affecting the growth of *C. violaceum*. AKG (2*S*)-3-*O*-(*cis*-13’-docosenyl)-1,2-propanediol was the best QS inhibitor (20 μM), while (2*S*)-3-*O*-(*cis*-9’-hexadecenyl)-1,2-propanediol was the least effective (795 μM). The results showed for the first time the QSI activity of this natural AKG series and suggest that AKGs could be promising candidates for further studies on preventing antimicrobial resistance.

## 1. Introduction

Antibiotics were discovered in the early 20th century and have been widely used in the treatment of bacterial infections. However, the indiscriminate and excessive usage of antibiotics has led to the development of antimicrobial resistance, which has steadily decreased the effectiveness of current antibacterial therapies [1,2]. Resistance mechanisms occur in almost all clinical isolates of bacteria and recurrent infections caused by persistent bacteria hamper the successful treatment of infections [3]. This situation highlights the urgency of identifying new therapeutic candidates and less toxic treatment targets. Consequently, considerable research has focused on developing novel strategies to control bacterial diseases [4]. Infectious diseases cause the death of more than 16 million people annually and at least 65% of these cases are linked to bacterial communities that proliferate by forming biofilms [5,6]. Biofilms consist of microorganisms that are attached to a substratum and embedded in a matrix of extracellular polymers that protects the cells from harsh environmental conditions, including disinfectant treatments and antibiotics [7]. Bacterial biofilms are developed by a cell communication process called quorum sensing (QS), through which Gram-negative and Gram-positive bacteria synchronize their behaviors in a cell density-dependent mode by a series of signal molecules termed autoinducers, thereby mediating the production and secretion of virulence traits. In general, QS is a regulatory mechanism that promotes the establishment of infection, expression of virulence factors, formation of biofilm and development of resistance [8,9]. Biofilms are involved in many clinical infections and during biofilm infection, simultaneous activation of both innate and acquired host immune responses may occur; however, these responses do not eliminate the biofilm pathogen but rather accelerate collateral tissue damage [10]. A strategy for controlling microbial infections involves the use of agents that inhibit bacterial pathogenicity (e.g., production of virulence factors and biofilm formation) rather than targeting growth-dependent mechanisms that will inevitably lead to the development of microbial resistance [11,12]. QS inhibitors do not kill bacteria or inhibit bacterial growth; rather, they quench QS-regulated pathogenic behaviors, which inhibits bacterial pathogenesis and does not easily induce antibiotic-resistant mutations [13,14]. The communication of individual cells is essential for the formation of biofilms; therefore, blocking this QS process is an important goal for the control of biofilm infections and represents a new method of combating antimicrobial resistance [15].

Efforts have been made to disrupt biofilms by inhibiting the QS system and many natural and synthetic molecules exhibiting quorum sensing inhibition (QSI) have been identified with potential therapeutic approaches [2]. However, natural products continue to be a prolific source of drugs [13,16] and the rich biodiversity in the marine environment suggests that it represents an enormous resource of novel therapeutic candidates with antibiofilm and QSI activities [17,18]. Marine sources, such as shark liver oil, contain high levels of alkylglycerols (AKGs), which are minor constituents of bacteria, protozoa, fungi, higher plants, animals and humans [19,20]. Natural AKGs called 1-*O*-alkyl-sn-glycerols are structurally glycerol ether lipids that occur as a mixture of alkyl chains of varying lengths and unsaturation levels and as pure enantiomers with an *S* configuration at the asymmetric carbon [21,22]. AKGs generally occur in their diacylated form as 1-*O*-alkyl-2,3-diacyl-sn-glycerols and they are also natural precursors of their derivatives ether phospholipids, which participate in the structure and function of cell membranes [20,23]. AKGs display a broad range of beneficial effects on human health and can be used to treat gastric ulcers and colon inflammation; stimulate hematopoiesis, immunological defense and vaccination efficiency; reduce tumor growth, metastasis, radiotherapy side effects, obesity and oxidative stress; increase therapeutic molecule permeability through the hematoencephalic barrier and sperm motility; and regulate cell differentiation and neuropathic pain [19,20,23,24,25,26,27,28]. Their activity spectrum also includes antifungal [29] and antibacterial properties [30,31,32,33,34,35].

In preliminary works to identify bioactive compounds from marine invertebrates [36], extracts from two soft coral *Eunicea* species and an AKG purified from them, which is known as batyl alcohol **8**, showed antibiofilm capacity against bacteria isolated from marine fouled surfaces [37,38]. Two more AKGs demonstrated growth inhibition of marine biofilm-forming bacteria, which are regarded as potential antifouling compounds [39,40]. In addition, a mixture of AKGs from *Eunicea* together with the AKG (2*S*)-3-*O*-dodecyl-1,2-propanediol **4** and (2*S*)-3-*O*-tetradecyl-1,2-propanediol **5** also showed QSI activity in *C. violaceum* ATCC 31532 [41]. Fifteen natural enantiopure AKGs were synthesized and they depicted promising ability against two biofilm-forming bacteria, clinical isolate *Staphylococcus aureus* 91 and *Pseudomonas aeruginosa* ATCC 15442 [42]. Thus, in the current study, we selected this natural AKG series to evaluate their antibiofilm activity against other clinical bacterial isolates, other reference strains and assess their QSI ability using *C. violaceum* ATCC 12472.

## 2. Results

### 2.1. Structures of Natural and Enantiopure Alkylglycerols

**Figure 1:**
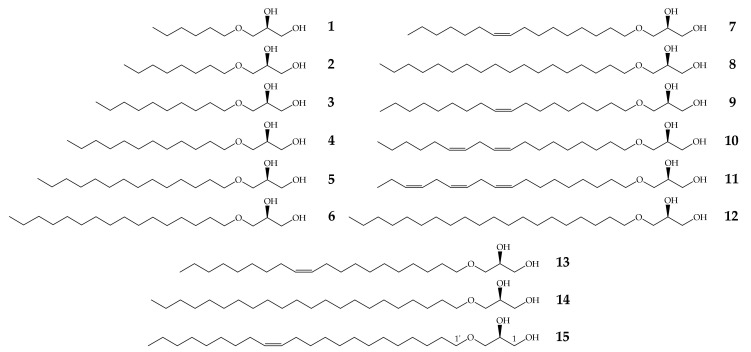
Structures of alkylglycerols used in this study.

The AKGs included in this study, **1–6**, **8**, **12** and **14**, are of saturated aliphatic chains, while AKGs **7**, **9**, **13** and **15** are mono-unsaturated, **10** is di-unsaturated and **11** is a tri-unsaturated AKG. They have a chain from 6 to 22 carbons atoms and between 176 to 400 amu (Figure 1).

### 2.2. Minimal Inhibitory Concentration (MIC) of Alkylglycerols

An initial in vitro screening was performed to evaluate the MIC of all AKGs (Figure 1) against the bacterial strains included in this study and the results are summarized in Table 1.

### 2.3. Alkylglycerols Ability to Inhibit of Biofilm Formation

**Figure 2:**
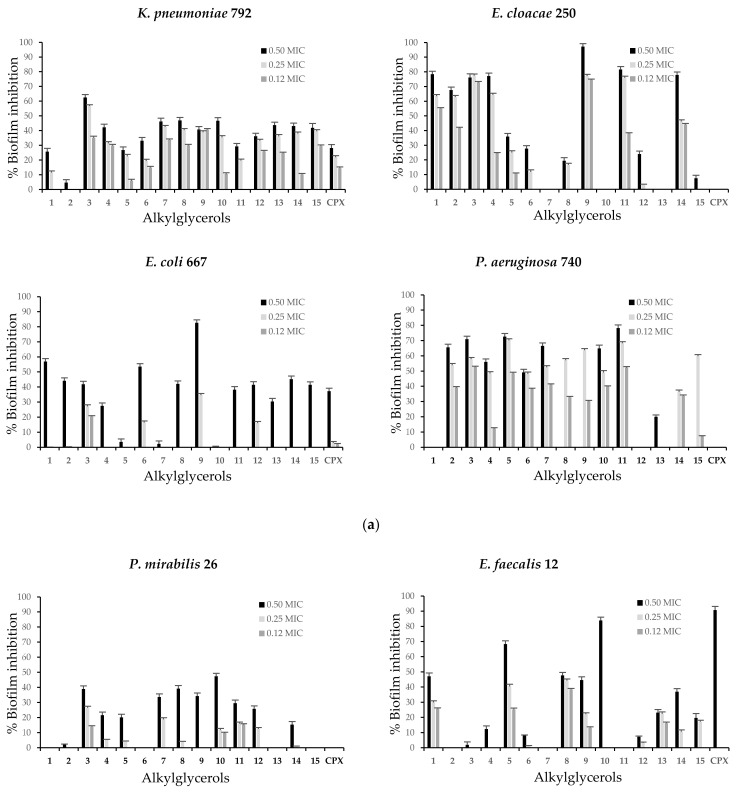

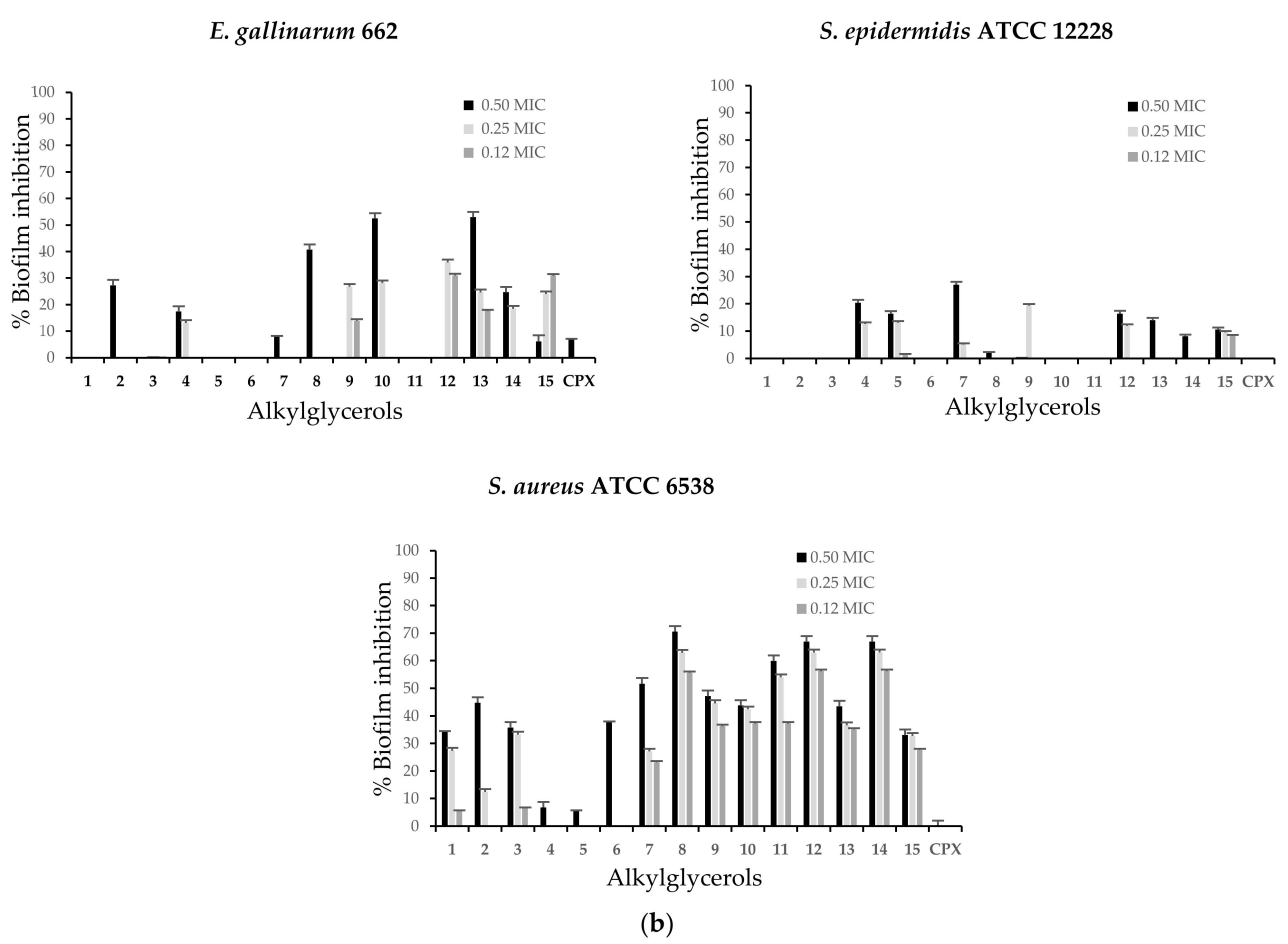
(**a**) Biofilm inhibition represented as percentages of enantiopure alkylglycerols against clinical bacterial isolates: *K. pneumoniae* 792, *E. cloacae* 250, *E. coli* 667 and *P. aeruginosa* 740 at different subinhibitory concentrations. (**b**) Biofilm inhibition represented as percentages of enantiopure alkylglycerols against clinical bacterial isolates: *P. mirabilis* 26, *E. faecalis* 12, *E. gallinarum* 662 and reference strains *S. epidermidis* ATCC 12228 and *S. aureus* ATCC 6538 at different subinhibitory concentrations. CPX was subject to the same assays. The results are expressed as the mean of three independent experiments and error bars represent the standard deviation. Biofilm formation was significantly reduced compared to untreated control biofilms of each of these bacteria at the tested concentrations of the compounds.

The clinical bacterial isolates and the reference strains were selected because of their biofilm-forming ability evidenced in this study. All examined AKGs were able to inhibit biofilm formation of most clinical isolates and reference strains and showed different capacities against each microorganism assayed. However, the inhibition intensity varied depending on the compound concentration (Figure 2a,b). The highest values of the biofilm inhibitory percentage were observed at AKGs concentrations corresponding to 0.50 MIC. Remarkable biofilm inhibition ratios of several AKGs above 50% were found for *E. cloacae* 250, *P. aeruginosa* 740 and *S. aureus* ATCC 6538, while a low response below 27% was found for *S. epidermidis* ATCC 12228. *K. pneumoniae* 792 and *S. aureus* ATCC 6538 were the only strains active against all AKGs.

AKG **9** showed a reduction in biofilm formation of all strains exhibiting the best antibiofilm activity and the highest inhibition ratios were observed for the Gram-negative clinical isolates, *E. cloacae* 250 (97.2%), *E. coli* 667 (82.6%) at 0.50 MIC and *P. aeruginosa* 740 (63.7%) at 0.25 MIC. AKG **11** showed great biofilm reduction for *E. cloacae* 250 (81.6%) and *P. aeruginosa* 740 (78.2%) at 0.50 MIC. The other AKGs were less effective.

*K. pneumoniae* 792 was inhibited by AKG **3** up to 62.5% and AKGs **7**, **8** and **10** by close to 46.5% at 0.50 MIC. In *E. cloacae* 250 in addition to AKGs **9** and **11**, AKGs **1**, **3**, **4** and **14** showed effectiveness and reached inhibition rates of approximately 76% at 0.50 MIC. Other compounds, such as **1** and **6**, decreased biofilm formation by *E. coli* 667 by up to 56.9% and AKGs **2**, **3**, **8**, **12**, **14** and **15** reached almost 45% at 0.50 MIC.

In *P. aeruginosa* 740, biofilm inhibition was also shown by AKGs **3** and **5** at up to 72.6% at 0.50 MIC and a lower response was observed with other compounds. At 0.50 MIC compounds **10** (47.3%), **3** and **8** were the most active against *P. mirabilis* 26. In the case of Gram-positive strains, *E. faecalis* 12 was most susceptible to AKGs **10**, **5** and **1** and an inhibition rate of 83.9% was found for **10**. In addition, for *E. gallinarum* 662 moderate inhibition by AKGs **13** (52.9%), **10** (52.4%) and **8** (40.7%) was observed at 0.50 MIC. For the reference strain *S. epidermidis* ATCC 12228, compounds **7** (27.0%) and **4** (20.3%) at 0.50 MIC exhibited low activity, whereas for *S. aureus* ATCC 6538 showed inhibition up to 70.6% for AKG **8** at 0.05 MIC and up to 66.9% for compounds **12** and **14**. In comparison, CPX, which is the antibacterial agent and subjected to same treatment, only inhibited biofilm formation of *E. faecalis* 12 up to 90.7%, *E. coli* 667 up to 37.2% and *K. pneumoniae* 792 up to 28.2% at 0.50 MIC.

### 2.4. Quorum Sensing Inhibition Activity of Natural Alkylglycerols

To determine the effect on the QSI activity using *C. violaceum* ATCC 12472 without interference from the antibacterial activity of the tested compounds, the AKGs were analyzed at subinhibitory concentrations. All AKGs were capable of inhibiting violacein production with varying degrees of action depending on the aliphatic chains on their structures (Table 2). Notably, the highest QSI activity was exhibited by AKG **15**, with a minimum concentration of 20 µM. To a lesser extent, compounds **3**, **4**, **5** and **10** showed relevant activity and reached a reduction in QS up to an inhibitory concentration of 135, 120, 109 and 92 µM respectivelly, followed by AKG **6** at a minimum concentration of 197 µM. In regard to the majority of AKGs, the potential of QSI was lower for **1**, **2**, **8**, **9**, **11**, **12**, **13** and **14** at a concentration varied from 312 to 709 µM, while *C. violaceum* ATCC 12472 was less sensitive for the inhibition of QS to AKG **7** (795 µM).

The bacterial viability of *C. violaceum* ATCC 12472 was also assessed for both the positive control and AKG-treated samples and the results in terms of colony-forming units/mL (CFU/mL) did not show significant differences between the groups. Therefore, the reduction in violacein production was attributed to the effect of the AKGs.

## 3. Discussion

Bacteria have developed multiple mechanisms to adapt to changes in the environment, especially the widespread use of antibiotics and these mechanics allow them to avoid adverse conditions and maintain their pathogenesis. One of the mechanisms is QS, a cell-to-cell communication method that depends on the cell density to control collective behavior [1,9]. With the regulation of QS, bacteria can form virulence factors such as toxins, motility, enzymes, proteins and biofilm development to survive in disadvantageous circumstances [2,8]. Biofilms are another critical reason for microbial resistance and they consist of bacteria embedded in a self-produced extracellular matrix of polysaccharides, fibrins, lipids, proteins and DNA that can reduce the effect of antibiotics [7,10,43]. Consequently, there is an urgent search for new candidates and less toxic treatments to control pathogenic resistant bacteria [3,4].

To identify the inhibitors of biofilm formation from bioactive extracts of two Caribbean soft corals of *Eunicea* genus, we isolated an AKG from these species called batyl alcohol **8**, which was the most active inhibitor [37,38]. This class of natural AKGs that had not been previously studied for antibiofilm activity was selected and great variety of their compounds were evaluated. AKGs are bioactive compounds found in low concentrations in marine sources, such as fishes, sponges and corals and at trace levels in plants and mammal, including humans [19,20,23,24,25,26,27,28]. In nature, they are enantiomerically pure constituents with configuration *S* at the stereogenic center [21,22], which motivated us to obtain several of their naturally occurring structures by enantiomeric synthesis [41].

In this study, fifteen previously synthesized enantiopure AKGs [42] were tested to determine their effect on the biofilm formation of seven clinical bacterial isolates and two reference strains and evaluate their QSI ability using *C. violaceum* ATCC 12472 at subinhibitory concentrations. These AKGs displayed weak antibacterial activity compared with CPX (MIC from 0.09 to 48.2 µM) against all strains at MIC values in the range of 27 to >1418 µM in a microtiter plate assay (Table 1, Figure 1). In this way, only saturated AKGs with an intermediate length of their aliphatic chain such as compounds **3**, **4** and **5** showed in vitro moderate antibacterial activity toward some Gram-positive strains, of them AKGs **4** and **5** were the most active against *S. aureus* ATCC 6538 (MIC of 30 and 27 μM). With less effectiveness against the most bacterial strains resulted the AKGs **2**, **6**, **7**, **9**, **10** and **11** between 99 and 1224 μM, while the AKGs, **1**, **8**, including **12**, **13**, **14** and **15**, the latter which have 20 or more carbon atoms at the aliphatic chain were much less active against all bacterial strains with a MIC from 624 to >1418 μM. Furthermore, the AKGs showed low bacteriostatic effects on the clinical isolates and reference strains because evaluation of the viability by seeding in fresh culture medium led to the recovery of each bacterial species (data not shown). The above is in accordance with other authors and our preliminary works [41,42]. In a previous study, the authors showed that racemic **4** (*rac*-**4)**, known as *rac*-1-*O*-dodecylglycerol, inhibited the growth of *Enterococcus faecium* and *Streptococcus mutans* primarily by stimulating autolysin activity and interfering with cell wall synthesis [30,31,32]. Racemic-**4** inhibits *S. aureus* and toxic shock syndrome toxin 1 production, although the mechanisms of action have not been characterized [35] and produces growth inhibition of members of two yeast genera, *Candida* and *Cryptococcus* [29]. AKGs **1** and **2** are able to prevent the growth of *Chlamydia trachomatis* [33], while AKG **8** show antimicrobial activity toward some marine bacterial species [34,38].

We found that all AKGs at subinhibitory amounts of 0.50, 0.25 and 0.12 MICs were capable of significantly inhibiting biofilm formation by up to 97.2% in both Gram-negative and Gram-positive clinical isolates and in the reference strains, although they had different efficiencies against each strain. (Figure 2a,b). This compound concentration-dependent antibiofilm activity is also dependent on the bacterial susceptibility of each strain to the respective structure. Consistent with the abovementioned findings, we previously observed that most of these AKGs reduced biofilm formation with different capacities against other two strains and the rates were 23.5–99.8% for the clinical isolate *S. aureus* 91 and 14.1–64.0% for *P. aeruginosa* ATCC 15442 [42]. Our additional results against these nine bacterial species considerably broaden the understanding of the antibiofilm abilities of AKGs.

The QSI activity of the AKGs in this work was explored by employing *C. violaceum* ATCC 12472 as a biological model and subinhibitory concentrations. All enantiopure AKGs significantly inhibited violacein production at minimum QS inhibitory concentrations in the range of 20 to 795 μM (Table 2). Surprisingly, among the tested compounds, the most potent QSI activity was noted for the highest molecular weight and unsaturated AKG, namely, (2*S*)-3-*O*-(*cis*-13’-docosenyl)-1,2-propanediol **15**, with a minimum concentration of 20 µM, while (2*S*)-3-*O*-(*cis*-9’-hexadecenyl)-1,2-propanediol **7** turned out to have the lowest QSI capacity (795 μM). In addition, AKGs **3**, **4**, **5** and **10** reached a reduction in QS at a minimum concentration of 135, 120, 109 and 92 µM, respectively. Neither AKG affected the growth of *C. violaceum* ATCC 12472. A compound that can to interfere with QS without affecting cell growth is considered a promising inhibitor [11,13,14]. The QSI effects reported in the current study support our preliminary results, where the disc diffusion assay in other strain, *C. violaceum* ATCC 31532, showed that AKG **4** exhibited the same minimum inhibition halo at 20 μg/disc as kojic acid, which is a positive control used as known inhibitor of QS systems, moreover, AKG **5** produced a minimum inhibitory halo at 50 μg/disc [41].

Although it was not possible to deduce a common relationship pattern between the structure and antibiofilm activity or between the structure and QSI activity, each strain positively responded with the aliphatic chain nature of the corresponding AKG. Some related natural molecules, such as fatty acids, are antibacterial agents at high concentrations and have great potential to attenuate microbial biofilm formation and virulence at low concentrations by modulating the QS systems without generating drug resistance [44]. Many studies have been performed to discover natural compounds that could combat pathogenic bacteria. Many natural products with antibiofilm activity have been identified from plants, microbes and marine life and they include various compounds classes, such as alkaloids, fatty acids, organosulfurs, cyclic compounds, phenolics, steroids, terpenoids and other aliphatic compounds. These compounds include: elligic acid glycosides, hamamelitannin, carolacton, skyllamycins, promysalin, phenazines, bromoageliferin, flustramine C, meridianin D and brominated furanones [18,45,46,47,48,49,50]. The most significant groups of plant-derived QS inhibitors belong to terpenes, terpenoids, phenylpropanoids, acid derivatives, diarylheptanoids, coumarins, flavonoids and tannins [46,51,52]. Microorganisms and marine organisms produce a range of molecules that showing remarkable QSI activity. These molecules belong to organic compounds classes: derived alkaloids, terpenes, peptides, polyketides, derived carbohydrates, sesterterpenes, halogenated furanones, cembranoids, halogenated alkaloids and phenolics and they include compounds, such as kojic acid, phenethylamides, aculenes, meleagrin, malyngamides, manoalides, hymenialdisin, betonicines, floridoside and cembrane diterpenoids [18,53,54].

Among the Gram-negative bacteria susceptible to AKGs in the present study, *K. pneumoniae* is a pathogenic bacterium that shows high rates of antibiotic resistance through biofilm formation [55]; *E. cloacae* is an emerging pathogen responsible for various diseases, including respiratory tract infections associated with biofilm formation [56]; *E. coli* pathogenic strains cause problematic biofilm infections and employ a furanosyl borate diester (AI-2)-based QS system to regulate two of its most studied phenotypes, virulence and biofilm formation [57]; another pathogen that has been well studied and is frequently associated with a wide range of severe infections is *P. aeruginosa*, which includes cystic fibrosis pneumonia and chronic obstructive pulmonary disease-related infections. The high level of *P. aeruginosa* pathogenicity is mostly controlled by QS, which regulates genetic expression, with 30% encoding virulence factor production and biofilm formation based on the release and sensing of the signal molecules homoserine lactones [6,8,9,11,57]; *P. mirabilis* is another opportunistic pathogen implicated in various human diseases of the respiratory tract and gastrointestinal tract and such infections are complicated by the unique ability of this bacterium to form crystalline biofilms that protect it from antibiotics and the host immune response [58]; among the most known Gram-negative bacteria, *C. violaceum* is a biosensor strain used for QS research that produces the pigment violacein in response to QS-regulated gene expression and it has been widely used to study the inhibition of QS [4,59]. Among Gram-positive bacteria, *E. faecalis* has become one of the most prevalent multidrug-resistant hospital pathogens and its QS system is closely related to biofilm development and virulence production [60]; *E. gallinarum* is less commonly identified in humans but is responsible for bloodstream, urinary tract and surgical wound infections and is resistant to many antibiotics [61]; the most important virulence factor of *S. epidermidis* is considered to be its ability to form biofilms on implanted biomaterials, these films are difficult to treat because of the increased resistance to both antibiotics and the human immune system [62]; in some cases, *S. aureus* causes a wide range of human infections on skin and soft tissues, as well as life-threatening pneumonia, bacteremia, osteomyelitis and toxic shock syndrome. *S. aureus* uses an autoinducing peptide signal to mediate QS and its pathogenicity depends on its ability to produce biofilms and virulence factors, such as different toxins, that contribute to invasion of the host and bacterial spread, [35,63].

In the current research, the differences in susceptibility among the bacteria tested to each AKG suggested that a large range of these structures with different molecular sizes, unsaturation and polarities have both antibiofilm and QSI activities, which result in much more effectiveness than their respective antibacterial effects. However, the most noteworthy information obtained from the present work revealed for the first time that this variety of natural AKGs are promising QS inhibitors. Since the target of QSI is bacterial virulence factors and bacterial biofilms, not viability, there is less chance that the bacteria will develop resistance to QS inhibitors [5,12,63]. For the above reasons, AKGs could be considered potential candidates to control pathogenic bacterial diseases and thus warrant further experiments involving other model systems to establish their action mechanisms and the extent of their efficacy against antimicrobial resistance.

## 4. Materials and Methods

### 4.1. General

#### 4.1.1. Screened Compounds

Fifteen AKGs previously synthesized in our laboratory in enantiomerically pure forms, such as the naturally occurring (Figure 1), were included in this study [42]: (2*S*)-3-*O*-hexyl-1,2-propanediol **1,** (2*S*)-3-*O*-octyl-1,2-propanediol **2**, (2*S*)-3-*O*-decyl-1,2-propanediol **3**, (2*S*)-3-*O*-dodecyl-1,2-propanediol **4**, (2*S*)-3-*O*-tetradecyl-1,2-propanediol **5**, (2*S*)-3-*O*-hexadecyl-1,2-propanediol **6**, (2*S*)-3-*O*-(*cis*-9’-hexadecenyl)-1,2-propanediol **7,** (2*S*)-3-*O*-octadecyl-1,2-propanediol **8**, (2*S*)-3-*O*-(*cis*-9’-octadecenyl)-1,2-propanediol **9,** (2*S*)-3-*O*-(*cis*,*cis*-9’,12’-octadecadienyl)-1,2-propanediol **10**, (2*S*)-3-*O*-(*cis*,*cis*,*cis*-9’,12’,15’-octadecatrienyl)-1,2-propanediol **11**, (2*S*)-3-*O*-(eicosyl)-1,2-propanediol **12**, (2*S*)-3-*O*-(*cis*-11’-eicosenyl)-1,2-propanediol **13**, (2*S*)-3-*O*-(docosyl)-1,2-propanediol **14** and (2*S*)-3-*O*-(*cis*-13’-docosenyl)-1,2-propanediol **15**.

#### 4.1.2. Bacterial Strains and Culture Conditions

The following bacterial strains were used during our antibacterial, antibiofilm and QSI experiments. The Gram-negative clinical isolates included *Klebsiella pneumoniae* 792, *Enterobacter cloacae* 520, *Escherichia coli* 667, *Pseudomonas aeruginosa* 740 and *Proteus mirabilis* 26. The Gram-negative reference strain *Chromobacterium violaceum* 12472 was donated by Prof. Catalina Arevalo from the Universidad Nacional de Colombia. The Gram-positive clinical isolates include *Enterococcus faecalis* 12 and *Enterococcus gallinarum* 662 and reference strains *Staphylococcus epidermidis* ATCC 12228 and *Staphylococcus aureus* ATCC 6538. The clinical strains were previously isolated from hospital patients and provided by the Hospital of Neiva (Huila), Hospital of Tunal, Hospital of Engativá and Universidad del Bosque (Bogotá) [41] and together with the other reference strains, they were obtained from our microbiology laboratory collection [41,42].

The strains were grown for 24 h at 37 °C in yeast extract malt extract dextrose agar (YMD, Merck, Darmstadt, Germany) and the inoculum was prepared in Mueller-Hinton broth (MHB, Merck), supplemented with glycerol (15% *w*/*v*) and kept at −20 °C until use. In addition, *C. violaceum* 12472 was freshly cultured for 24 h at 22 °C in lysogeny broth (LB, Merck) supplemented with kanamycin 100 μg/mL (Merck) before use.

### 4.2. Determination of the Minimum Inhibitory Concentration of Screened Compounds

The MIC of AKGs was determined in a microbroth dilution assay according to the Clinical and Laboratory Standards Institute guidelines with slight modifications [42]. Briefly, the inoculum (100 μL, 1 × 10^4^ CFU/mL) was mixed with AKG dissolved in 3.6% dimethyl sulfoxide (DMSO, Merck) solution in water to final concentrations of 250, 125, 62.5, 31.25, 15.63, 7.81, 3.91, 1.95, 0.98 or 0.49 μg/mL in MHB, with up to 200 μL added to each well. All experiments were conducted using a maximum of 0.9% (*v*/*v*) DMSO in medium. The plates were incubated at 37 °C for 24 h under aerobic conditions, while *C. violaceum* 12472 was incubated at 22 °C. Ciprofloxacin (CPX, Merck) was used as the standard antibacterial agent (stock concentrations of 64 μg/mL). MIC was defined as the lowest concentration of AKGs to completely inhibit bacterial growth, which was indicated by a lack of visual turbidity. An inoculated well with solvent in culture medium (positive control) and a well containing only medium (negative control) were included. All experiments were performed in triplicate.

### 4.3. Effectiveness of Alkylglycerols on the Inhibition of Biofilm Formation

The ability of AKGs to prevent biofilm formation of bacterial strains included in this study was evaluated on 96-well polystyrene microplates (Techno Plastic Products, Trasadingen, Switzerland) according to the method described by Stepanović et al. [64], with some modifications [42]. Briefly, 100 μL of the bacterial strain grown in MHB medium supplemented with 1% glucose was inoculated in each well to 10^5^ CFU/mL in the presence of 100 µL subinhibitory concentrations of each AKG dissolved in aqueous solution of DMSO, equivalent to 0.50, 0.25 and 0.12 of MIC or of the highest concentration assayed during the MIC evaluation (250 μg/mL). The DMSO concentrations were not toxic to strains assayed and did not affect biofilm formation (data not shown). After incubation for 48 h at 37 °C, the wells were washed three times with 200 µL of water to remove planktonic bacteria and dried. The remaining bacteria that adhered to the surface of the wells were fixed with 200 µL of methanol for 15 min. Then, the wells were emptied, air dried and subsequently stained with 200 µL of 2% crystal violet solution (Merck) for 5 min, followed by washing with water, drying and solubilizing the stain using 200 µL of 33% acetic acid for 10 min. The absorbance of each well was measured at 570 nm on a microplate reader (xMark Bio-Rad, Hercules, CA, USA). Additionally, CPX was subject to the same essay. The values are expressed as the percent biofilm inhibited in comparison to the untreated control biofilm (positive control). All tests were performed in triplicate.

### 4.4. Screening for Quorum Sensing Inhibition Activity

The QSI ability of the AKGs was evaluated in vitro in 96-well microtiter plates by a *C. violaceum* ATCC 12472 biosensor bioassay [4], as previously described with some modifications [41]. Briefly, preinoculum cultured in LB supplemented with kanamycin was adjusted to 1 × 10^9^ CFU/mL at 600 nm. Then, 100 μL was added to each well, followed by 100 μL of AKGs dissolved in DMSO aqueous solution at subinhibitory concentrations of each compound or of the highest MIC assessed (250 μg/mL) and the plate was incubated for 48 h at 22 °C. Thus, the QSI activity was evaluated as the minimum quantity in μg/mL of sample required to inhibit violacein pigment and it was established by the appearance of a colorless and opaque well and did not have an effect on bacterial growth. The sample solvent that had no antibacterial activity served as the negative control and *C. violaceum* 12472 grown in the presence of the same amount of solvent without AKG was used as the positive control. To determine the effect of the presence of each AKG on the growth of the biosensor, a colony count was performed in each assay. All experiments were carried out with three replicates.

### 4.5. Statistical Analysis

All antibiofilm experiments were performed in triplicate and the results were expressed as the means ± standard deviation and calculated using statistical analyses of random uncertainties and rejection of data [65]. MIC and QSI data are representative of three independent experiments and the minimum concentration is expressed as the mean value.

## 5. Conclusions

AKGs are known bioactive compounds with weak to moderate antibacterial activity. Here, fifteen natural enantiopure AKGs were tested to determine their effect on biofilm development by seven clinical bacterial isolates and two reference strains as well their effect on the QSI activity in *C. violaceum* ATCC 12472 by microtiter plate assays. Ciprofloxacin as the standard antibiotic underwent the same antibiofilm assay. At subinhibitory concentrations, the highest biofilm inhibition rates (%) exhibited for all AKGs were influenced in a concentration-dependent manner and each AKG acted individually against the bacterial isolates, reaching rates up to 97.2%. In addition, AKGs displayed minimum QS inhibitory concentrations at different levels but did not affect the growth of *C. violaceum*. (2*S*)-3-*O*-(*cis*-13’-docosenyl)-1,2-propanediol **15** was the most effective AKG against QSI (20 µM), while (2*S*)-3-*O*-(*cis*-9’-hexadecenyl)-1,2-propanediol **7** was the least active (795 µM). Additional to the novelty of antibiofilm data, the results showed for the first time the QSI activity of this natural AKG series, which indeed, suggests that AKGs constitute a class of promising candidates for further studies on preventing antimicrobial resistance.

## Figures and Tables

**Table 1:** Minimal inhibitory concentration values (µM) of the examined alkylglycerols.

Strains	Alkylglycerols ^a^									
	CPX ^b^	2	3	4	5	6	7	9	10	11
*K. pneumoniae* 792	0.09	>1224	>1076	>960	>867	>790	>795	>730	>734	>738
*E. cloacae* 250	48.2	>1224	>1076	>960	>867	395	>795	>730	>734	>738
*E. coli* 667	0.09	>1224	>1076	>960	>867	>790	>795	>730	>734	>738
*P. aeruginosa* 740	1.51	>1224	>1076	>960	>867	>790	>795	>730	>734	>738
*P. mirabilis* 26	0.09	>1224	1076	>960	>867	>790	>795	>730	>734	>738
*C. violaceum* ATCC 12472	0.18	>1224	269	>960	>867	>790	>795	730	>734	>738
*E. faecalis* 12	0.51	>1224	538	119	433	>790	199	365	734	>738
*E. gallinarum* 662	48.2	>1224	>1076	119	>867	>790	>795	>730	>734	>738
*S. epidermidis* ATCC 12228	0.39	>1224	538	119	108	>790	795	>730	734	>738
*S. aureus* ATCC 6538	0.18	612	67	30	27	>790	99	>730	367	369

^a^ Alkylglycerols **1**, **8**, **12**, **13**, **14** and **15** had a MIC values >1418, >726, >671, >675, >624 and >627 µM respectively for all tested bacterial strains. All experiments were performed with a maximum of 0.9% (*v*/*v*) DMSO in MHB medium and it did not interfere with bacterial growth. ^b^ CPX = ciprofloxacin. CPX was the antibacterial agent used as the standard. Values are the means of triplicate determinations.

**Table 2:** Minimum quorum sensing inhibitory concentration (µM) of alkylglycerols against *C. violaceum* ATCC 12472.

Values. µM ^a^	**Alkylglycerols**														
	**1**	**2**	**3**	**4**	**5**	**6**	**7**	**8**	**9**	**10**	**11**	**12**	**13**	**14**	**15**
	709	611	135	120	109	197	795	363	365	92	369	335	337	312	20

^a^ Minimum quantity μM of compound required to inhibit violacein pigment. Values represent the means from three independent experiments.
